# The Predictive Capacity of the 3-Meter Backward Walk Test for Falls in Older Adults: A Case–Control Analysis

**DOI:** 10.3390/jfmk10020154

**Published:** 2025-04-30

**Authors:** Luis Polo-Ferrero, Javier Torres-Alonso, María Carmen Sánchez-Sánchez, Ana Silvia Puente-González, Fausto J. Barbero-Iglesias, Roberto Méndez-Sánchez

**Affiliations:** 1 Department of Nursing and Physiotherapy, University of Salamanca, 37007 Salamanca, Spain; javiertorres@usal.es (J.T.-A.); csanchez@usal.es (M.C.S.-S.); silviapugo@usal.es (A.S.P.-G.); fausbar@usal.es (F.J.B.-I.); ro_mendez@usal.es (R.M.-S.); 2 Institute of Biomedical Research of Salamanca (IBSAL), 37007 Salamanca, Spain

**Keywords:** older adults, aging, frailty, falls, fall risk, backward walking, 3 m backward walk, functional assessment, geriatric evaluation, physical performance

## Abstract

**Background**: The early detection of fall risk in older adults is crucial for prevention. This study assessed the 3-Meter Backward Walk Test (3m-BWT) as a predictor of falls. **Methods**: A retrospective observational case–control study was conducted with 483 community-dwelling participants (mean age 76.3 ± 6.5 years), including 101 individuals with a history of falls in the previous 12 months. A standardized battery of functional assessments was applied. **Results**: Significant differences were observed between fallers and non-fallers across all functional variables (*p* < 0.001), with fallers demonstrating slower performance on the 3m-BWT (6.8 ± 3.4 s vs. 5.1 ± 1.3 s). The 3m-BWT showed moderate correlations with Short Physical Performance Battery, 5-repetition Sit-to-Stand, gait speed, and 4-Square Step Test, and a moderate-to-strong correlation with Timed Up-and-Go (r = 0.632), even after adjusting for age, sex, and BMI. Although the 3m-BWT exhibited superior discriminative ability compared to other tests (AUC = 0.655), its predictive power in isolation remains limited. The optimal cut-off point was identified at 5.5 s (sensitivity: 59.5%; specificity: 68.6%), while a threshold of <3.5 s yielded high sensitivity (98%) but low specificity, supporting its use in fall risk screening. **Conclusions**: These findings support the integration of the 3m-BWT as a complementary tool within comprehensive geriatric assessments, particularly in contexts requiring high sensitivity. Given the multifactorial nature of falls, combining the 3m-BWT with other clinical evaluations and fall history is recommended to enhance risk stratification and inform preventive strategies.

## 1. Introduction

Aging is a complex process involving structural, functional, and psychological changes, leading to cellular and organ deterioration, reduced responsiveness, and increased disease susceptibility [1,2]. This process contributes to geriatric syndromes, particularly frailty, which is prevalent among older adults and heightens the demand for social and healthcare services [3,4]. Frailty results from declines in physiological reserve, increasing vulnerability to stressors, and is characterized by symptoms such as exhaustion, low physical activity, unintentional weight loss, weakness, and slow gait [5,6]. It is a precursor to disability and a significant risk factor for falls in older adults [7,8].

Falls are common among older adults, with about 30% of those over 65 experiencing a fall each year, and the rate increases after age 75 [9,10]. The risk of recurrence within a year is approximately 66% [11]. Falls in older adults are caused by a combination of intrinsic and extrinsic factors, including underlying diseases, medications, psychological factors, and environmental conditions [12]. Between 10% and 25% of falls result in serious injury, making them the leading cause of fatal injury and trauma-related hospital admissions in this population [9,13]. In the United States, the average cost of hospital treatment for a fall injury exceeds USD 30,000 [14], underscoring the importance of fall prevention and early risk detection in older adults.

Fall prevention efforts have utilized medical records, questionnaires, and functional tests to assess patient risk, with such tests being essential in comprehensive geriatric evaluations. The Timed Up and Go Test (TUG) and 5 Times Sit-to-Stand Test (5STS) are two of the most widely used functional tests predictive of falls and are widely supported in the literature. However, none have demonstrated high discriminative accuracy in isolation [15]. Specifically, a TUG time greater than 12 s and a 5STS time greater than 15 s are commonly used cut-off points to indicate increased fall risk, while gait speed (GS) values below 0.7 to 1.0 m/s, depending on the source, and Short Physical Performance Battery (SPPB) scores below 8 have also been associated with a higher likelihood of falling in older adults [16,17,18,19,20,21]. Other tests, such as Hand Grip Strength (HG) and the 4-Square Step Test (4SST), have also been linked to frailty and fall risk, though without widely accepted cut-off thresholds for predicting falls [22,23]. These tests primarily assess forward motion or turning, whereas walking backward, which demands greater neuromuscular and proprioceptive control [24], is less studied [25]. Thus, exploring functional tests that assess backward walking and their predictive power for falls in older adults is necessary.

The 3-Meter Backward Walk Test (3m-BWT) is a clinical assessment that measures the ability to walk backward. It is an innovative functional measure that has been the subject of studies since 2019 [26]. A recent systematic review, which covered studies published between 2019 and 2023, evaluated the measurement properties of the test. The results confirmed its validity and reliability in patients with various clinical conditions, including community-dwelling older adults, and demonstrated its sensitivity to change, providing normative data for these populations. However, the authors noted that many of the included studies had limited sample sizes (less than 100 participants), which restricts the generalizability of the findings. Therefore, they recommended further research involving larger samples to obtain more robust evidence regarding the predictive validity of the 3m-BWT in the assessment of fall risk [27]. This study aims to address that gap by evaluating the discriminative capacity of the 3m-BWT and establishing optimal cut-off points with adequate sensitivity and specificity. A 3.5 s threshold has been proposed in older adults, which may enhance its clinical applicability in fall risk screening [26].

By comparing fallers and non-fallers, this study aims to identify the functional characteristics that most clearly differentiate these groups, thereby enhancing the clinical utility of the 3m-BWT in fall risk assessment. It also seeks to provide more robust and accurate information on the predictive ability of the 3m-BWT in older adults, using a larger sample than previous studies. By strengthening the evidence on its predictive validity, we seek to support the integration of this test as a potential tool for identifying fall risk within comprehensive geriatric assessments and to contribute to the development of effective fall prevention strategies.

## 2. Materials and Methods

### 2.1. Study Design

A retrospective observational case–control study was conducted among older adults to establish reference values for the 3m-BWT in predicting falls. This study is part of a research project approved by the Ethics Committee for Drug Research of the Salamanca Health Area (Protocol Code: PI 2024061702), conducted in accordance with the Declaration of Helsinki [28], and registered on ClinicalTrials.gov on 23 May 2024 (Registration No.: NCT06434857). This study also adheres to the CONSORT 2010 guidelines [29].

### 2.2. Study Population

Participants were recruited annually through the Geriatric Revitalization Program (GRP) coordinated by the University of Salamanca, a community-based initiative aimed at promoting healthy aging. Recruitment was conducted through open calls in local media and community centers, and all participants enrolled voluntarily. Inclusion criteria comprised community-dwelling adults aged 60 years and older who were able to provide informed consent, complete functional and physical assessments, and follow verbal instructions adequately. Cognitive status was screened through a brief clinical interview conducted by trained personnel; individuals with suspected cognitive impairment or a prior diagnosis of dementia were excluded. Additional exclusion criteria included the presence of central nervous system disorders associated with neurological deficits (e.g., Parkinson’s disease, stroke), a history of major surgery or traumatic injury in the previous six months, and any musculoskeletal or cardiorespiratory condition contraindicating exercise. Individuals with visual, auditory, or sensory impairments that could compromise test performance and those with clinically diagnosed orthostatic hypotension were not included. The use of medication was not a criterion for exclusion, and pharmacological profiles were not analyzed in the present study. Participants were classified as cases or controls based on self-reported history of one or more falls within the 12 months prior to assessment.

### 2.3. Assessment and Outcomes

The evaluation was conducted under standardized and controlled conditions at the Research Unit of the Faculty of Nursing and Physiotherapy, University of Salamanca. For this study, the recruitment period took place between May and August 2024, and all testing sessions were conducted during the second half of September 2024. A pre-trained research team with over three years of experience in geriatrics was responsible for data collection. Each test was consistently administered by the same researcher throughout the study to ensure procedural uniformity and minimize inter-rater variability. Although multiple researchers participated in the evaluation process, each was assigned a specific test and conducted only that test across all participants. All assessments followed a standardized internal protocol (Table S1). To minimize bias, participants were anonymized using coded identifiers, and group allocation (fallers vs. non-fallers) was determined based on self-reported falls within the previous 12 months. Prior to participation, all individuals received detailed verbal and written information and provided written informed consent.

During the structured personal interview, sociodemographic variables (e.g., age and sex), relevant medical history, and selection criteria were collected. Body composition was assessed using bioelectrical impedance analysis (TANITA BC-418), recording weight, height, body mass index (BMI; weight/height^2^), appendicular skeletal muscle mass (ASM), and skeletal muscle mass index (SMI; ASM/height^2^).

Functional performance was assessed using the 3m-BWT as the main test, alongside other established functional tests commonly used to predict falls, and evaluated in this study based on their respective cut-off points. For the 3m-BWT, participants stood with their backs to a marked line and were instructed to walk backward until both feet had crossed a second marked line. They were allowed to look back but not to run [26]. The test was performed three times, and the best time was recorded using a smartphone stopwatch.

In addition to the 3m-BWT, this study included the TUG, which measures the time needed to rise from a chair, walk 3 m, turn around, return, and sit down; the 5STS, which assesses lower limb strength through five consecutive sit-to-stand repetitions; the GS, calculated from a 4 m walk at usual pace; and the SPPB, a composite score based on balance, gait speed, and chair rise performance. The HG test evaluates upper limb strength using a dynamometer, and the 4SST assesses dynamic balance through timed multidirectional stepping. Full protocols for all tests are available in Supplementary Table S1.

### 2.4. Sample Size

The sample size was determined using the GRANMO tool version 8, tailored for case–control studies. Given that the prevalence of falls among older adults is approximately 30%, a 3:1 ratio between controls and cases was anticipated [9]. To achieve an alpha risk of 0.05 and a statistical power greater than 0.8 in a bilateral contrast, 101 cases (fallers) and 353 controls (non-fallers) were needed to detect a minimum Odds Ratio of 2, accounting for an estimated 5% loss to follow-up.

### 2.5. Statistical Analysis

The data were analyzed using IBM SPSS Statistics 28.0, employing descriptive measures and frequencies with a significance level of 0.05. The normality of the data was verified using box plots and the Kolmogorov–Smirnov test. A descriptive analysis of the baseline characteristics was conducted for the faller and non-faller groups. The Chi-square test was employed for categorical variables with a normal distribution, while the McNemar test was used for those with a non-normal distribution. Quantitative variables were analyzed with the *t*-test for independent samples when they were normally distributed; otherwise, the Mann–Whitney U-test was used.

The correlation analysis examined the relationship between the 3m-BWT, other functional variables that predict falls (TUG, 5STS, GS, SPPB), and others related to frailty status (HG and 4SST) using the Pearson correlation coefficient when the data were normally distributed and the Spearman coefficient when they were not. In addition, a partial correlation analysis (r_p_) was performed, controlling for the possible effect of age, sex, and BMI. Correlations were classified as weak (0.1–0.3), moderate (0.4–0.6), and strong (0.7–1.0), following Cohen’s classification [30].

The area under the curve (AUC) of the receiver operating characteristic (ROC) curve was calculated to assess the discriminative ability of the functional tests in identifying falls. AUC values were interpreted according to established criteria: 0.5–0.6 (fail), 0.6–0.7 (poor), 0.7–0.8 (acceptable), 0.8–0.9 (excellent), and >0.9 (outstanding) [31]. Sensitivity and specificity were determined at different cut-off points on the ROC curve, as described in the scientific literature. A detailed analysis of the 3m-BWT test at various cut-off points was performed to identify the optimal value for detecting falls in older adults, evaluating its sensitivity and specificity. For each cut-off point, sensitivity, which reflects the model’s ability to correctly identify positive cases, and specificity, which assesses its ability to identify negative cases, were calculated. In addition, discriminative capacity was quantified using the Youden Index (J), providing a comprehensive measure of test accuracy at each threshold.

## 3. Results

### 3.1. Participant Characteristics

In this study, 483 older adults were included with no participant loss, achieving the expected case–control proportions. Table 1 presents demographic and anthropometric data for fallers (*n* = 101) and non-fallers (*n* = 382). Non-parametric tests were used due to the non-normal distribution of variables.

No statistically significant differences were observed in age between fallers (77.6 ± 6.8 years) and non-fallers (75.9 ± 6.4 years; *p* = 0.150). Although a significant difference in sex distribution was identified (*p* < 0.001), the absolute difference was minimal (5.2%) and likely of limited clinical relevance. Similarly, no significant differences were found in body weight (*p* = 0.151) or height (*p* = 0.610). However, when analyzing body composition variables, a statistically significant difference was observed in the body mass index (BMI; *p* = 0.038) and skeletal muscle mass index (SMI; *p* < 0.001), with fallers showing higher BMI and lower SMI values, indicating a potential imbalance between fat and lean mass components.

Functional tests showed significant differences between fallers and non-fallers. Fallers took longer in the 3m-BWT (6.8 ± 3.4 s vs. 5.1 ± 1.3 s; *p* < 0.001), 5STS (11.4 ± 4.6 s vs. 9.5 ± 2.2 s; *p* < 0.001), and especially in TUG (10.1 ± 3.3 s vs. 8.6 ± 1.8 s; *p* < 0.001) tests, demonstrating the effectiveness of these tests in identifying fall risk. Additional significant differences were also observed in GS (1.07 ± 0.26 m/s vs. 1.20 ± 0.3 m/s; *p* < 0.001), SPPB (10.9 ± 1.8 vs. 11.6 ± 0.9; *p* < 0.001), HG (21 ± 5.4 kg vs. 22.6 ± 6.2 kg; *p* = 0.018), and 4SST (7.4 ± 2.4 s vs. 6.4 ± 1.5 s; *p* < 0.001).

### 3.2. Correlation of 3m-BWT with Functional Variables

A simple correlation analysis (r) and a partial correlation analysis (rₚ) adjusted for age, sex, and BMI were performed. The 3m-BWT showed a weak correlation with HG (r = −0.199; 95% CI: −0.283, −0.112; r_p_ = −0.168), suggesting only a slight inverse association between faster 3m-BWT and higher HG, even when adjusted for age, sex, and BMI. Moderate correlations were identified with the SPPB (r = −0.511, 95% CI: −0.574, −0.442; r_p_ = −0.477), 5STS (r = 0.528, 95% CI: 0.460, 0.589; r_p_ = 0.505), GS (r = −0.517, 95% CI: −0.579, −0.448; r_p_ = −0.469), and 4SST (r = 0.596, 95% CI: 0.547, 0.644; r_p_ = 0.571), indicating that better functional performance on these measures is associated with faster 3m-BWT times, even after adjustment for sex, age, and BMI. A correlation between moderate and strong was observed with the TUG (r = 0.632, 95% CI: 0.575, 0.682; r_p_ = 0.581). All of these correlations were statistically significant (*p* < 0.001), highlighting the significant association of the 3m-BWT with several functional tests and emphasizing its value in assessing fall risk in older adults, even when controlling for the effect of age, sex, and BMI (Table 2).

### 3.3. Discriminative Capacity of Functional Tests

**Figure 1:**
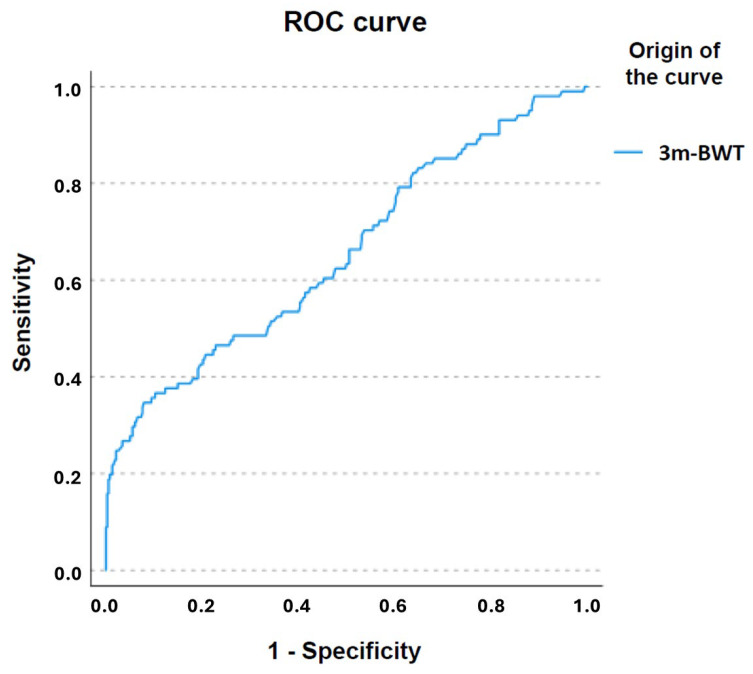
ROC curve of the 3m-BWT for identifying falls in older adults.

Regarding the discriminative capacity of functional tests in older adults who experience falls compared to those who do not, significant results were observed in all variables (*p* = 0.001), except for HG (*p* = 0.18; AUC = 0.424; 95% CI 0.363–0.484) (Table 3). The findings indicate that the 3m-BWT is the most effective test for identifying falls, with an AUC of 0.655 (95% CI: 0.592–0.719) and a *p*-value of 0.001 (Figure 1). However, this AUC still reflects limited discriminative ability, and its clinical use as a standalone predictor should be considered with caution.

The TUG demonstrated the second highest discriminative ability, with an AUC of 0.637 (95% CI: 0.573–0.699). The 5STS had an AUC of 0.636 (95% CI: 0.573–0.699), and the 4SST showed an AUC of 0.630 (95% CI: 0.566–0.694). The SPPB, a composite measure of physical performance, exhibited an AUC of 0.415 (95% CI: 0.349–0.482), indicating a moderate capacity to discriminate fallers, with a *p*-value of 0.013. In contrast, the HG test demonstrated limited discriminatory power, with an AUC of 0.424 (95% CI: 0.363–0.484) and a non-significant *p*-value of 0.18. The GS had the lowest discriminative capacity among the significant tests, with an AUC of 0.362 (95% CI: 0.303–0.422) and a *p*-value of 0.001.

### 3.4. Sensitivity and Specificity of Various Cut-Off Points

Various cut-off points of the 3m-BWT were analyzed to detect fall risk in older adults. The lowest cut-off points (3.0–4.5 s) showed high sensitivity (99.0% to 79.2%) but low specificity (2.1% to 37.7%), reflecting high fall detection but with numerous false positives and limited discriminative ability (J = 0.011 to 0.169). As the cut-off time increased, sensitivity decreased (46.5% at 6.0 s to 26.7% at 7.5 s), while specificity improved significantly (50.0% to 95.5%). The optimal cut-off point, with a low discriminative ability, was observed at 5.5 s (J = 0.280), balancing sensitivity (59.5%) and specificity (68.6%). Thus, in order to consider the predictive value, it is more optimal to consider the cut-off point with the highest sensitivity (3.5 s) and thus be able to identify people who may be at higher risk of falling. However, these results highlight the possibility of adjusting the cut-off point depending on the clinical context and objective. The full data are presented in Table 4.

## 4. Discussion

### 4.1. Main Findings

In this study, we evaluated the 3m-BWT as a practical and alternative functional assessment tool to predict fall risk in older adults. By analyzing data from 483 participants, we demonstrated its potential for identifying individuals at a high risk of falls, offering a simple yet effective option for clinical and community settings. The proportion of fallers in our sample (18.95%; *n* = 101) was slightly lower than previously reported (30%) [9]. Our results do not align with previous studies reporting an increased incidence of falls with advancing age [32,33], as no statistically significant age differences were found between fallers and non-fallers in our sample. However, we did confirm the association between a lower SMI and higher fall risk, consistent with previous research [34,35]. Significant differences in functional variables such as the TUG, 5STS, GS, SPPB, HG, and 4SST indicate that poorer performance in these components—reflected by slower times, lower strength, or reduced scores—is associated with increased fall risk. Among these, the 3m-BWT showed the largest difference between fallers and non-fallers, suggesting it may offer superior discriminative ability. However, despite being the best-performing test in our study, its overall discriminative capacity remains modest, and it should not be used as a standalone tool for fall prediction. These findings underscore the need for interventions focused on maintaining muscle mass and functionality to prevent falls in older adults, consistent with prior research [36].

The 3m-BWT reveals a significant difference of 1.71 s between fallers and non-fallers, which is statistically notable since the minimum detectable change in this population is 1.52 s [37]. This suggests that the 3m-BWT could be a valid tool for identifying and predicting fall risk, as it detects greater time differences in those who fall compared to those who do not. The 3m-BWT shows moderate correlations with other functional tests used for fall detection, such as the 5STS, SPPB, GS, and 4SST. It has a particularly strong relationship with the TUG, the most commonly used fall predictor [38]. Despite the different movements emphasized by the TUG and 3m-BWT, studies report similar correlations between them [27]. These findings support the potential use of the 3m-BWT as an additional fall prediction tool, as walking backward challenges postural control systems [24].

### 4.2. Clinical Implications

Backward walking with assistance from a parallel has been shown to be an important functional predictor of falls, surpassing even forward walking speed or the TUG in independent community-dwelling older adults [39,40]. These findings have been supported by performing the same movement unassisted by the 3m-BWT in our study. Although the 3m-BWT demonstrated the highest discriminative capacity among the tests evaluated in this study, its AUC of 0.655 still reflects poor overall discriminative ability. Therefore, it should not be used as a standalone tool to predict fall risk in older adults. Instead, its value lies in its potential as a complementary screening measure, especially in settings where high sensitivity is prioritized. For example, a cut-off point below 3.5 s shows high sensitivity but low specificity, making it useful to identify individuals at higher risk of falling, despite the risk of false positives. Conversely, specific populations such as those with Parkinson’s disease or multiple sclerosis may require adjusted thresholds, as indicated in previous research [41,42,43]. However, it is important to note that the different cut-off values explored in this study (e.g., 3.5 s and 5.5 s) have not yet been validated across specific clinical subgroups or in prospective designs. Future research should aim to confirm the predictive validity of these thresholds in relation to incident falls in diverse populations. Our findings support the integration of the 3m-BWT within a broader battery of functional assessments to help detect mobility limitations, rather than relying on it in isolation. These results are not surprising, as other commonly used tests, such as the TUG, SPPB, GS, HG, or 4SST, also do not show adequate discriminative ability to predict falls in older adults when used in isolation [44,45,46,47]. Further research is needed to develop a comprehensive fall risk assessment tool that can accurately identify common and individual risk factors. Falls are multifactorial, suggesting the need to explore other associated factors that may not influence performance on a balance test [44].

In settings where it is crucial to detect the highest number of individuals at risk, a cut-off point of 3.5 s identifies 98% of positive cases, albeit with a high false positive rate. This result reinforces previous findings, where the same cut-off point was reported but with a lower sensitivity (74%) than in our study [26]. If the aim is to find a more balanced cut-off point, we propose adjusting it to 5.5 s (0.72 m/s), taking advantage of the larger sample size. This threshold has sufficient sensitivity (59.5%) and specificity (68.6%), achieving balanced values comparable to previous studies, reducing false positives, and improving accuracy [25,26].

When interpreting the results, it is important to acknowledge this study’s limitations and consider recommendations for future research. The sample may not be fully representative of the general population, as it excludes institutionalized or hospitalized individuals, potentially affecting the generalizability of the findings. Additionally, this study did not account for all factors influencing falls, such as environmental, physiological, and psychological factors. Key aspects of physical performance, including stride length, proprioception, and attention, which might impact test results, were also not assessed. Falls were identified based on participants’ self-reports over the past year, which could be affected by memory issues or biases, and whether the individual had fallen one or more times was not recorded.

### 4.3. Limitations, Recommendations, and Future Perspectives

To address these limitations, future research should include more representative samples across various ages, genders, and health statuses, including institutionalized and hospitalized individuals. It should also be noted that participants in this study were recruited from a voluntary healthy aging program, which may have introduced a selection bias toward a more functional and motivated population. This may limit the generalizability of the findings to more frail, institutionalized, or hospitalized populations. Longitudinal studies are needed to evaluate the test’s effectiveness over time, track predictive changes in risk variables, and measure the impact of preventive interventions. Moreover, future studies should distinguish between single and recurrent falls, as this may improve the clinical relevance of the 3m-BWT and allow for more detailed analyses. Although partial correlations were adjusted for age, sex, and BMI, other potential confounders—such as comorbidities, medication use, cognitive status, and physical activity levels—were not controlled for. This represents an important limitation, and future studies should consider multivariate analyses that account for these variables to strengthen the validity of the findings. Additionally, relevant functional domains such as proprioception, cognitive function, dual-task gait performance, and reaction time—which are known to influence fall risk—were not evaluated in this study. It should also be acknowledged that the sample included a higher proportion of non-fallers than fallers, which could affect the generalizability of the group comparisons; however, this distribution is expected in community-dwelling older adult populations, where the prevalence of falls typically ranges from 20% to 30%. These omissions should be acknowledged as limitations, and future research is encouraged to incorporate these measures to enhance the comprehensiveness of fall risk assessments.

The results suggest that older adults who complete the 3m-BWT in less than 3.5 s are less likely to have a history of falls, while the risk appears to increase significantly above this time. However, as this is a cross-sectional study, these results should not be interpreted as predictive of future fall risk. Although the 5.5 s point offers a reasonable balance, its limitations highlight the need for further research in diverse populations to optimize predictive accuracy. Furthermore, the 3m-BWT should not be considered as a sole diagnostic tool but as part of a comprehensive geriatric assessment, combining it with other instruments and considering factors such as history of falls, individual characteristics, and other functional tests [44,45,46,47]. This will allow for a more accurate and personalized assessment of fall risk. Developing personalized fall risk protocols with specific interventions and ongoing follow-up could further enhance fall prevention efforts.

## 5. Conclusions

The findings of this study support the potential of the 3m-BWT as a complementary functional tool in the assessment of fall risk in older adults, highlighting its superior discriminatory ability compared to widely used tests such as the TUG, GS, and SPPB. However, its limited sensitivity and specificity underscore that it should not be used as a sole method, given the multifactorial nature of falls.

Test execution time emerges as a relevant indicator: Values below 3.5 s suggest minimal risk, while exceeding 5.5 s could signal greater vulnerability, albeit with predictive limitations. These results reinforce the need to integrate the 3m-BWT within a multidimensional approach, combining it with clinical assessments, falls history, and other functional tests to improve risk stratification.

Future research should explore the optimization of cut-off points, their validity in heterogeneous populations, and their interaction with other risk factors. In the meantime, the application of personalized protocols, accompanied by targeted interventions and continuous monitoring, could enhance more effective preventive strategies in the geriatric population. The 3m-BWT thus represents a promising advance, but its clinical utility will depend on a contextualized and holistic implementation.

## Figures and Tables

**Table 1:** Comparison of characteristics between non-fallers and fallers.

Variable	Non-Fallers (*n* = 382)	Fallers (*n* = 101)	*p*-Value
Female, (n, %)	328 (85.9%)	92 (91.1%)	<0.001
Age (years)	75.9 ± 6.4	77.6 ± 6.8	0.150
Height (m)	1.54 ± 0.08	1.54 ± 0.06	0.610
Weight (kg)	64.7 ± 11.6	66.9 ± 11.6	0.151
BMI (kg/m^2^)	27.2 ± 4.3	28.3 ± 4.6	0.038
SMI (kg/m^2^)	7.5 ± 0.9	6.2 ± 0.9	<0.001
3m-BWT (s)	5.1 ± 1.3	6.8 ± 3.4	<0.001
TUG (s)	8.6 ± 1.8	10.1 ± 3.3	<0.001
5STS (s)	9.5 ± 2.2	11.4 ± 4.6	<0.001
GS (m/s)	1.20 ± 0.3	1.07 ± 0.26	<0.001
SPPB	11.6 ± 0.9	10.9 ± 1.8	<0.001
HG (Kg)	22.6 ± 6.2	21 ± 5.4	0.018
4SST (s)	6.4 ± 1.5	7.4 ± 2.4	<0.001

Values expressed as means ± standard deviation (SD) and frequencies (n, %).

**Table 2:** Correlation of 3m-BWT with functional variables predictors of falls.

Functional Variables	r (CI 95%)	r_p_	*p*-Value
TUG ^a^	0.632 (0.575, 0.682)	0.581	<0.001
5STS ^a^	0.528 (0.460, 0.589)	0.505	<0.001
GS ^a^	−0.517 (−0.579, −0.448)	−0.469	<0.001
SPPB ^a^	−0.511 (−0.574, −0.442)	−0.477	<0.001
HG ^a^	−0.199 (−0.283, −0.112)	−0.168	<0.001
4SST ^a^	0.596 (0.547, 0.644)	0.571	<0.001

Values expressed as correlation coefficients and the confidence interval (r CI 95%), partial correlation (age, sex, and BMI), and *p*-value (*p*). ^a^: Spearman correlation coefficient. *p*-value of r.

**Table 3:** Global discriminative capacity of functional tests to identify falls in older adults.

Functional Variable	AUC	95% CI for AUC	*p*-Value
3m-BWT	0.655	0.592–0.719	<0.001
TUG	0.637	0.573–0.702	0.011
5STS	0.636	0.573–0.699	<0.001
GS	0.362	0.303–0.422	<0.001
SPPB	0.415	0.349–0.482	0.013
4SST	0.630	0.566–0.694	<0.001
HG	0.424	0.363–0.484	0.180

The values express the discriminatory capacity of the model according to the AUC, its confidence interval (95% CI), standard error, and *p*-value.

**Table 4:** Discriminative capacity of falls at different cut-off points of the 3m-BWT.

Cut-Off Points	Sensitivity (%)	Specificity (%)	Youden’s Index (J)
3.0 s	99.0	2.1	0.011
3.5 s	98.0	9.8	0.079
4.0 s	89.1	22.8	0.119
4.5 s	79.2	37.7	0.169
5.0 s	63.4	50.0	0.134
5.5 s	59.5	68.6	0.280
6.0 s	46.5	76.4	0.230
6.5 s	37.6	86.1	0.237
7.0 s	34.7	92.1	0.268
7.5 s	26.7	95.5	0.222

The values in real numbers represent the sensitivity, specificity, and discriminatory capacity (J) of different cut-off points.

## Data Availability

The data sets used and/or analyzed during the present study are available from the corresponding author upon request.

## Supplementary Materials

The following supporting information can be downloaded at https://www.mdpi.com/article/10.3390/jfmk10020154/s1, Table S1: Standardized measurement of the result variables.

## Abbreviations

The following abbreviations are used in this manuscript:

3m-BWT	3-Meter Backward Walk Test
4SST	4-Square Step Test
5STS	5 Times Sit-to-Stand Test
ASM	Appendicular Skeletal Muscle Mass
AUC	Area Under the Curve
BMI	Body Mass Index
GRP	Geriatric Revitalization Program
GS	Gait Speed
HG	Hand Grip Strength
ROC	Receiver Operating Characteristic
SMI	Skeletal Muscle Mass Index
SPPB	Short Physical Performance Battery
TUG	Timed Up and Go Test
