# Supplementary material for: The Predictive Capacity of the 3-Meter Backward Walk Test for Falls in Older Adults: A Case–Control Analysis
Source: J Funct Morphol Kinesiol. 2025 Apr 30;10(2):154. doi: 10.3390/jfmk10020154 (PMC12101408; doi:10.3390/jfmk10020154)
Supplement: Supplementary file 1 [file jfmk-10-00154-s001.zip › jfmk-3597798-supplementary.pdf]

Table S1. Standardized measurement of the result variables.

<table>
<tr><td>Weight, ASM, SMI, and BMI</td><td colspan="3">All participants had their weight reduced by 1 kg for clothing. All subjects were properly hydrated before the test. The surface of the BIA was wetted to improve handling, and all participants were measured completely barefoot.</td></tr>
<tr><td>3m-BWT</td><td colspan="3">All participants start with their backs to a marked line. They are told to walk backwards until they have cleared the next mark with both feet. They are allowed to look back if they wish. Three measurements are allowed, with the best being selected.</td></tr>
<tr><td>TUG</td><td colspan="3">Consists of measuring the time it takes an individual to get up from a chair, walk 3 metres, turn around and return to a sitting position, all at normal speed and without assistance. The test was carried out twice and the best mark was chosen, taking care not to use the hands to get up.</td></tr>
<tr><td>GS</td><td colspan="3">The patient stands a few metres before the line where the time starts and is instructed to walk at his or her usual speed until three metres after the line where the time stops. The distance over which it was calculated was 4 metres. It was performed three times and the best value was chosen.</td></tr>
<tr><td>5xSTS</td><td colspan="3">Performed using an armless folding chair with a seat height of 17 inches (43.2 cm). The time starts when the patient initiates the first squat from the seated position and ends when the patient returns to a seated position after performing all 5 squads.</td></tr>
<tr><td>HG</td><td colspan="3">To perform the measurement in a standardised manner, all participants were placed in a standing position, elbow flexed at 90º and neutral pronosupination and were told: "Squeeze with all your strength for 3 seconds! The test was performed alternately with the left and right hand and the mean was obtained as the valid value.</td></tr>
<tr><td>4SST</td><td colspan="2">The participant stands in quadrant 1 of an imaginary cross made up of four squares. He must move in the order 2, 3, 4, 1 and then back (1, 4, 3, 2) as quickly as possible without touching the markers. The total time is measured using a stopwatch.</td><td>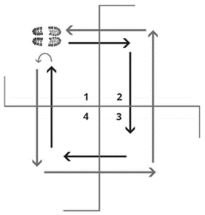</td></tr>
<tr><td rowspan="3">SPPB</td><td rowspan="3">This is a rapid, objective physical function test with three tests. It is scored from 0 to 12 points, each section can be scored up to 4 points according to the marks obtained.</td><td>Balance</td><td>Held for 10s with feet together. Subsequently in a semitandem position. The last test had to be held for more than 10 seconds in tandem position.</td></tr>
<tr><td>Gait Speed</td><td>Same</td></tr>
<tr><td>5STS</td><td>Same.</td></tr>
</table>

3m-BWT, 3-meter Backward Walk Test; 4SST, Four Square Step Test; 5xSTS, Five Times Sit-to-Stand Test; ASM, Appendicular Skeletal Muscle Mass; BMI, Body Mass Index; GS, Gait Speed; HG, Hand Grip Strength; SMI, Skeletal Muscle Index; SPPB, Short Physical Performance Battery; TUG, Timed Up and Go Test.
